# Role of the 5-HT_2A_ Receptor in Acute Effects of LSD on Empathy and Circulating Oxytocin

**DOI:** 10.3389/fphar.2021.711255

**Published:** 2021-07-13

**Authors:** Friederike Holze, Isidora Avedisian, Nimmy Varghese, Anne Eckert, Matthias E. Liechti

**Affiliations:** ^1^ Clinical Pharmacology and Toxicology, Department of Biomedicine and Department of Clinical Research, University Hospital Basel and University of Basel, Basel, Switzerland; ^2^ Department of Pharmaceutical Sciences, University of Basel, Basel, Switzerland; ^3^ Psychiatric University Hospital, University of Basel, Basel, Switzerland; ^4^ Transfaculty Research Platform Molecular and Cognitive Neuroscience, University of Basel, Basel, Switzerland

**Keywords:** LSD, empathy, oxytocin, emotion processing, ketanserin, 5-HT2A receptor, psychedelic

## Abstract

The psychedelic lysergic acid diethylamide (LSD) has experienced a revival in research, including clinical trials that evaluate LSD-assisted psychotherapy. LSD induces perceptual alterations and influences emotion processing in ways that may support psychotherapy. Here, we investigated the effects of LSD on emotional empathy and mediating role of the serotonin 5-hydroxytryptamine-2A (5-HT_2A_) receptor by administering 25, 50, 100, and 200 µg LSD alone and 200 µg LSD combined with pretreatment with the 5-HT_2A_ receptor antagonist ketanserin (40 mg) using a placebo-controlled, double-blind, random-order, crossover design in 16 healthy subjects. The Multifaceted Empathy Test (MET) was used to assess the effects of LSD on emotional empathy. Plasma oxytocin levels were also measured. LSD dose-dependently increased implicit and explicit emotional empathy, with the highest 200 µg LSD dose having a significant effect compared with placebo. The 200 µg dose of LSD also moderately increased plasma oxytocin levels compared with placebo. Ketanserin reduced the LSD-induced elevations of oxytocin but not the LSD-induced increases in emotional empathy. These findings confirm that LSD enhances empathy, and this effect may be partially independent of its primary action on 5-HT_2A_ receptors to induce subjective psychedelic effects. In contrast, LSD-induced oxytocin release may depend on 5-HT_2A_ receptor stimulation, which is consistent with the psychedelic effect of LSD. Further studies are needed to investigate whether LSD may also enhance empathy and potentially produce therapeutic effects in patients who have deficits in empathy and impairments in social functioning.

## Introduction

The prototypical psychedelic lysergic acid diethylamide (LSD) is experiencing a revival in psychiatric research. Possible medical benefits of the substance are currently investigated in Phase 2 trials in patients with anxiety clinicaltrials.gov no. NCT03153579; (Gasser et al., 2014), depression (clinicaltrials.gov no. NCT03866252), and cluster headache clinicaltrials.gov no. NCT03781128; (Gasser et al., 2014; Carhart-Harris et al., 2015; Dolder et al., 2015; Schmid et al., 2015; Carhart-Harris et al., 2016a; Strajhar et al., 2016; Holze et al., 2020; Holze et al., 2021). The mechanism by which LSD produce potential therapeutic effects in different disorders is not well understood. The primary target for LSD to produce its acute psychedelic effects in humans appears to be the serotonin 5-hydroxytryptamine-2A (5-HT_2A_) receptor because the typical acute subjective effects of LSD can be nearly completely blocked by pretreatment with the 5-HT_2A_ receptor antagonist ketanserin (Preller et al., 2017; Holze et al., 2021). Ketanserin alone possesses no psychoactive properties but may result in dry mouth, nasal congestion, tiredness, and reduced blood pressure (Persson et al., 1991). However, LSD also binds with high affinity to 5-HT_1A_ and dopamine receptors (Rickli et al., 2016). Many downstream mechanisms have been proposed to be involved in the potential therapeutic effects of psychedelics, including LSD, such as an increase in neuroplasticity, reflected by increases in brain-derived neurotrophic factor (Ly et al., 2018; Hutten et al., 2020), alterations of brain network connectivity (Carhart-Harris et al., 2016b; Mueller et al., 2018; Mueller et al., 2017b), socioemotional effects (Schmid et al., 2015), and related changes in emotion processing (Dolder et al., 2016; Mueller et al., 2017a). LSD has been shown to produce empathogenic and prosocial effects (Dolder et al., 2016; Schmid et al., 2015). Specifically, LSD acutely increased feelings of subjective well-being, happiness, closeness to others, openness, and trust (Dolder et al., 2016; Schmid et al., 2015), impaired the recognition of sad and fearful faces in the Face Emotion Recognition Task, enhanced emotional empathy in the Multifaceted Empathy Test (MET), and increased prosocial behavior in the Social Value Orientation Test. Such empathogenic and prosocial effects were otherwise typically reported for the prototypical empathogen 3,4-methylenedioxymethamphetamine (MDMA; (Hysek et al., 2014; Bershad et al., 2016; Dolder et al., 2018), which mainly releases serotonin and oxytocin (Dumont et al., 2009; Hysek et al., 2012; Hysek et al., 2014; Francis et al., 2016) and is also investigated in substance-assisted psychotherapy. The findings indicate that LSD and MDMA may have overlapping socioemotional effect properties. Oxytocin is thought to play a critical role in emotion processing. Intranasal oxytocin administration increased emotional empathy, trust, and positive communication and enhanced emotion recognition (Kosfeld et al., 2005; Di Simplicio et al., 2009; Ditzen et al., 2009; Hurlemann et al., 2010). Interestingly, LSD also increased plasma oxytocin levels, although only at a high dose (Schmid et al., 2015; Holze et al., 2020) and to a lesser extent than MDMA (Holze et al., 2020). The effects of different low to high doses of LSD on empathy have not yet been investigated. Unclear is whether the empathy-enhancing and oxytocin-releasing effects of LSD are mediated by 5-HT_2A_ receptors.

Therefore, the present study investigated the effects of LSD (0, 25, 50, 100, and 200 μg) on the MET. Additionally, the 5-HT_2A_ receptor antagonist ketanserin or placebo was administered prior to the highest 200 μg LSD dose to examine whether the effects of LSD on emotional empathy and circulating plasma oxytocin levels are mediated by 5-HT_2A_ receptors. The subjective and autonomic effects of LSD and pharmacokinetics of LSD that were investigated in the present study were reported elsewhere (Holze et al., 2021). However, subjective effects (“any drug effect”) and plasma LSD concentrations are reported herein as peak responses and at 6 h after LSD administration when the MET was performed.

## Materials and Methods

### Study Design

The study used a double-blind, placebo-controlled, crossover design with six experimental test sessions to investigate the responses to (i) placebo, (ii) 25 µg LSD, (iii) 50 µg LSD, (iv) 100 µg LSD, (v) 200 µg LSD, and (vi) 200 µg LSD 1 h after ketanserin administration (40 mg) as reported in detail elsewhere (Holze et al., 2021). Block randomization was used to counterbalance the different dosing conditions. The washout periods between sessions were at least 10 days. The study was conducted in accordance with the Declaration of Helsinki and International Conference on Harmonization Guidelines in Good Clinical Practice and approved by the Ethics Committee of Northwest Switzerland (EKNZ) and Swiss Federal Office for Public Health. All of the subjects provided written consent before participating in the study, and they were paid for their participation. The study was registered at ClinicalTrials.gov (NCT03321136). Other effects of LSD that were assessed in this study were reported previously (Holze et al., 2021).

### Participants

Sixteen healthy subjects (eight men and eight women; mean age ± SD: 29 ± 6.4 years; range: 25–52 years) were recruited by word of mouth or an advertisement that was posted on the web market platform of the University of Basel. Mean body weight was 69 kg. Exclusion criteria were age <25 years or >65 years, pregnancy (urine pregnancy test at screening and before each test session), personal or family (first-degree relative) history of major psychiatric disorders (assessed by the Semi-structured Clinical Interview for Diagnostic and Statistical Manual of Mental Disorders, 4th edition, Axis I disorders by a trained psychiatrist), the use of medications that may interfere with the study medications (e.g., antidepressants, antipsychotics, and sedatives), chronic or acute physical illness (e.g., abnormal physical exam, electrocardiogram, or hematological and chemical blood analyses), tobacco smoking (> 10 cigarettes/day), lifetime prevalence of illicit drug use > 10 times (except for Δ^9^-tetrahydrocannabinol), illicit drug use within the last 2 months, and illicit drug use during the study period (determined by urine drug tests). The participants were asked to consume no more than 10 standard alcoholic drinks/week and have no more than one drink on the day before the test sessions. Additionally, the participants were not allowed to drink xanthine-containing liquids after midnight on the study day. The drug use history of the participants was reported elsewhere (Holze et al., 2021). Drug of abuse tests that were performed for each subject once during screening and once during the study were negative.

### Study Procedures

The study included a screening visit, six 25-h test sessions (each separated by at least 10 days), and an end-of-study visit. The sessions were conducted in a calm standard hospital room equipped with a standard hospital bed for the participant and a desk and a chair for the investigator. The room had an adjoining balcony, which participants were allowed to access after peak effects had subsided. Only one research subject and one investigator were present during each test session. Participants were allowed to bring their own music and to bring occupation for the time after effects had subsided or for placebo days (e.g. book, laptop, games etc.). Blindfolds were provided upon request. The test sessions began at 7:45 AM. A urine sample was taken to verify abstinence from drugs of abuse, and a urine pregnancy test was performed in women. The subjects then underwent baseline measurements. Ketanserin (40 mg) or placebo was administered at 8:00 AM. LSD or placebo was administered at 9:00 AM. Standardized lunches and dinners were served at 1:30 PM and 6:00 PM, respectively. The subjects were never alone during the first 16 h after drug administration, and the investigator was in a room next to the subject for up to 24 h. The subjects were sent home the next day at 9:15 AM.

### Study Drug

LSD base (> 99% purity; Lipomed AG, Arlesheim, Switzerland) was administered as an oral solution that was produced according to good manufacturing practice in units that contained 100 or 25 µg LSD in 1 ml of 96% ethanol (Holze et al., 2019; Holze et al., 2021). Ketanserin was obtained as the marketed drug Ketensin (20 mg, Janssen-Cilag, Leiden, Netherlands) and encapsulated with opaque capsules to ensure blinding. Placebo consisted of identical opaque capsules that were filled with mannitol. A double-dummy method was used. The subjects received two capsules and two solutions in each session: (i) two placebo capsules and placebo/placebo solutions, (ii) two placebo capsules and 25 µg LSD/placebo solutions, (iii) two placebo capsules and 25 µg LSD/25 µg LSD solutions, (iv) two placebo capsules and 100 µg LSD/placebo solutions, (v) two placebo capsules and 100 µg LSD/100 µg LSD solutions, and (vi) two ketanserin capsules and 100 µg LSD/100 µg LSD solutions.

### Measures

*Multifaceted Empathy Test.* The MET is a reliable and valid task that assesses cognitive and emotional aspects of empathy (Dziobek et al., 2008). The MET has been shown to be sensitive to the effects of oxytocin (Hurlemann et al., 2010), MDMA (Hysek et al., 2014; Kuypers et al., 2014; Schmid et al., 2014), psilocybin (Preller et al., 2015), and LSD (Dolder et al., 2016). The computer-assisted test consisted of 40 photographs that showed people in emotionally charged situations. To measure emotional empathy, the subjects were asked to rate how much they were feeling for an individual in each scene (i.e., explicit emotional empathy) and how much they were aroused by each scene (i.e., implicit emotional empathy) on a 1–9 point scale. The latter rating provides an inherent additional assessment of emotional empathy, which is considered to reduce the likelihood of socially desirable answers. To assess cognitive empathy, the participants were required to infer the mental state of the subject in each scene and indicate the correct mental state from a list of four responses. Cognitive empathy was defined as the percentage of correct responses relative to total responses. The three aspects of empathy were each tested with 20 stimuli with positive valence and 20 stimuli with negative valence, resulting in a total of 120 trials. The MET was performed 6 h after LSD administration.

*Subjective mood.* The visual analog scale (VAS) for “any drug effect” was assessed repeatedly over 24 h. The VAS was presented as a 100-mm horizontal line (0 ± 100%), marked from “not at all” on the left to “extremely” on the right. The questionnaire was also administered 6 h after drug administration, immediately before the MET was performed.

*Oxytocin concentrations.* Oxytocin concentrations in blood plasma were measured using an oxytocin enzyme-linked immunosorbent assay kit (ENZO Life Sciences, Ann Arbor, MI, United States) according the manufacturer’s protocol (Holt-Lunstad et al., 2008). Plasma was diluted in a 2:1 ratio in 1.5% TFA-H_2_O and centrifuged at 17,100 × *g* for 15 min at 4°C. The supernatant was collected and then loaded onto an Oasis PRiME HLB 96-well plate with 30 mg sorbent (Waters, Milford, MA, United States) and washed with 0.1% TFA-H_2_O. The sample was then eluted with 95% acetonitrile + 5% of a 0.1% TFA-H_2_O solution. The elute was collected and dried in an Eppendorf centrifugal vacuum concentrator (model no. 5301, Eppendorf AG, Hamburg, Germany). For the measurements, the residue was reconstituted in assay buffer. The reconstituted samples, standards, and controls were plated on a goat anti-rabbit immunoglobulin G microtiter plate and incubated with oxytocin conjugate at 4°C overnight. The plate was then washed, and the substrate was added. The reaction was stopped after 1 h, and the plate was read at 405 nm. Oxytocin concentrations were determined from a standard curve that was calculated from a four-parameter logistics curve fit. Blood samples for oxytocin concentration determination were taken 1, 3, and 8 h after LSD or placebo administration.

*LSD concentrations.* Blood samples for the analysis of plasma LSD levels were collected repeatedly in lithium heparin tubes. Plasma concentrations of LSD were determined by ultra-high-performance liquid chromatography tandem mass spectrometry with a lower limit of quantification of 5 pg/ml as reported elsewhere (Holze et al., 2019; Holze et al., 2021).

### Statistical Analyses

Dose-response effects of LSD (25, 50, 100, and 200 µg) on emotional empathy measures were assessed using repeated-measures analysis of variance (ANOVA), with drug as the within-subjects factor, followed by the Tukey *post hoc* test based on significant main effects. Separate ANOVAs were applied to assess the effect of ketanserin on the LSD response, with drug (LSD alone, LSD + ketanserin, and placebo) as the within-subjects factor, followed by the Tukey *post hoc* test based on significant main effects. The data were analyzed using Statistica 12 software (StatSoft, Tulsa, OK, United States). The criterion for significance was *p* < 0.05.

## Results

### Empathy

**FIGURE 1:**
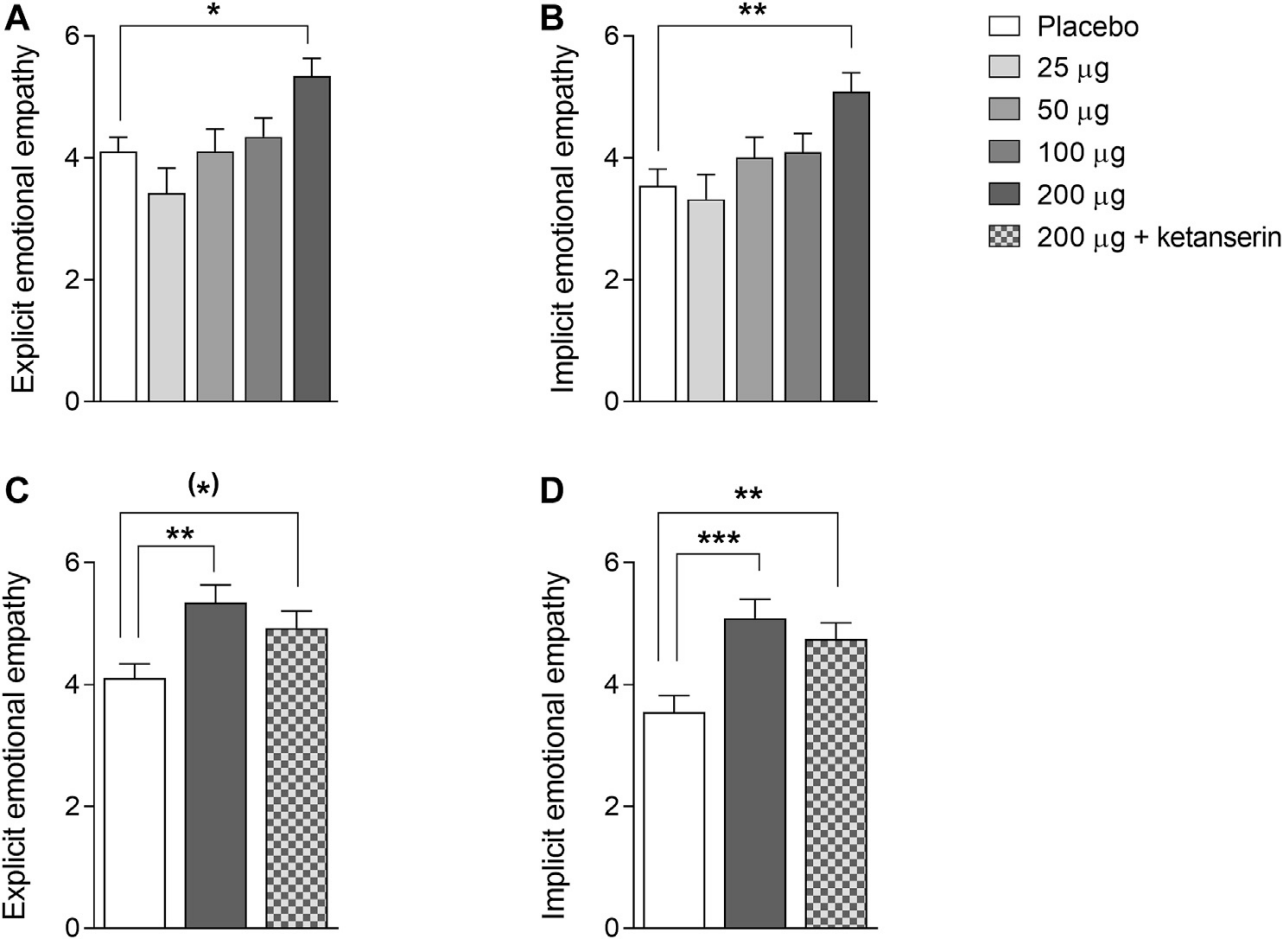
Acute effects of LSD on emotional empathy on the Multifaceted Empathy Test. LSD dose-dependently increased explicit **(A)** and implicit **(B)** emotional empathy compared with placebo, with the 200 µg LSD dose producing significant effects. The increases in explicit **(C)** and implicit **(D)** empathy that were induced by the 200 µg LSD dose were only slightly reduced by ketanserin pretreatment compared with placebo pretreatment and remained elevated compared with placebo. The data are expressed as the mean ± SEM in 16 subjects ^(^*^)^ *p* < 0.1, *p < 0.05, **p < 0.01, ***p < 0.01.

The effects of LSD on explicit and implicit emotional empathy are shown in Figure 1. There were significant main effects of LSD on explicit and implicit emotional empathy ratings (*F*_4,44_ = 4.64, *p* < 0.01, and *F*_4,44_ = 4.82, *p* < 0.01, respectively), indicating that LSD dose-dependently and significantly increased both aspects of emotional empathy. Only the highest 200 µg dose of LSD significantly affected explicit (*p* < 0.05) and implicit (*p* < 0.01) empathy scores compared with placebo. LSD did not alter cognitive empathy (data not shown).

We then examined the effect of pretreatment with ketanserin on the LSD-induced increase in empathy. There were significant main effects of drug on explicit and implicit emotional empathy ratings (*F*_2,28_ = 4.53, *p* < 0.05, and *F*_2,28_ = 8.56, *p* < 0.01, respectively). The *post hoc* tests showed that both LSD and LSD + ketanserin increased explicit (*p* < 0.05 and *p* = 0.09, respectively) and implicit (*p* < 0.001 and *p* < 0.01, respectively) empathy compared with placebo. Ketanserin did not significantly reduce the LSD-induced increase in empathy (Figure 1).

### Subjective Drug Effects

**FIGURE 2:**
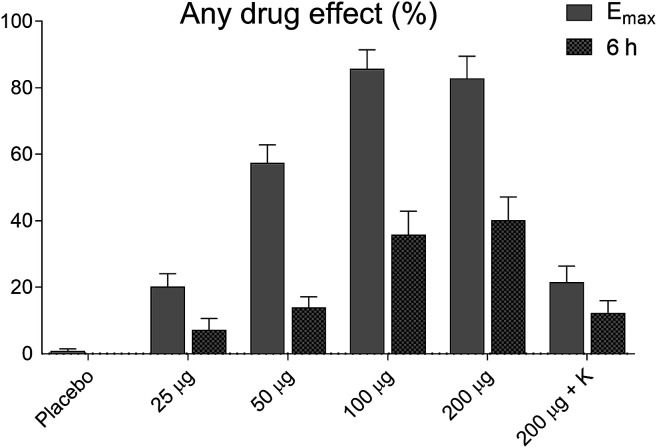
Acute subjective effects of LSD. LSD dose-dependently induced subjective effects, measured as “any drug effects,” on the VAS. The data show the subjective effects as maximal response and at 6 h after drug administration when the empathy test was performed. The data are expressed as the mean ± SEM in 16 subjects.

The subjective effects of LSD on “any drug effect,” assessed by the VAS, are shown in Figure 2. LSD dose-dependently produced subjective effects that reached half-maximal levels 6 h after LSD administration when the MET was administered.

### Oxytocin Concentrations

**FIGURE 3:**
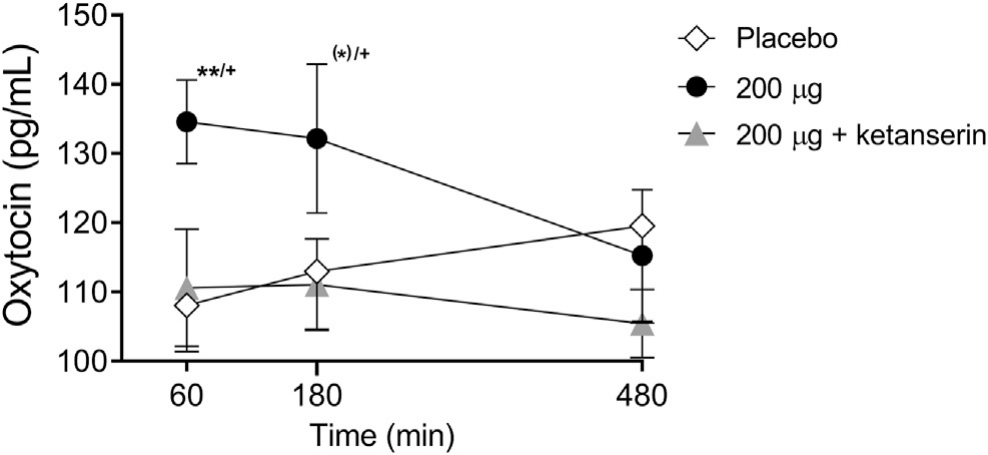
Effects of LSD on circulating oxytocin. LSD alone significantly increased plasma oxytocin concentrations 60 min after administration compared with placebo. LSD did not increase oxytocin levels after ketanserin pretreatment compared with placebo pretreatment. The data are expressed as the mean ± SEM in 16 subjects ^(^*^)^ *p* < 0.1, **p < 0.01, compared with placebo; ^+^ *p* < 0.05, compared with LSD + ketanserin.

There were significant main effects on oxytocin release 1 and 3 h after drug intake (*F*_2,28_ = 7.00, *p* < 0.01, and *F*_2,28_ = 4.21, *p* < 0.05, respectively). The *post hoc* tests showed that only LSD alone increased oxytocin levels 1 and 3 h after drug intake (*p* < 0.01 and *p* = 0.06, respectively) compared with placebo and at 1 and 3 h after drug intake (*p* < 0.01 and *p* < 0.05, respectively) compared with the LSD + ketanserin (Figure 3). Thus, ketanserin effectively prevented the LSD-induced increase in circulating oxytocin.

### Plasma Drug Levels

Plasma LSD concentrations were 2.2 ± 1.0 ng/ml (mean ± SD) 6 h after administration of the 200 µg dose and 2.5 ± 0.9 ng/ml 6 h after the administration of 200 µg LSD + ketanserin, which was at the time when the empathy task began. For comparison, E_max_ concentrations after 200 µg LSD and 200 µg LSD + ketanserin were 3.9 ± 0.8 and 4.4 ± 0.8 ng/ml, respectively. The full pharmacokinetic data on LSD from this study were published previously in detail (Holze et al., 2021).

## Discussion

LSD enhanced explicit and implicit emotional empathy and increased plasma oxytocin concentrations at a dose of 200 µg. The results confirmed previous findings (Schmid et al., 2015; Dolder et al., 2016). Consistent with past study findings, both explicit and implicit emotional empathy were enhanced by LSD and only by the highest 200 µg dose and not by doses of 100 µg or lower (Dolder et al., 2016). Similarly, the psychedelic psilocybin and empathogen MDMA have been shown to increase emotional empathy on the MET (Hysek et al., 2014; Kuypers et al., 2014; Preller et al., 2015), indicating that all of these psychoactive substances that are currently being investigated to assist psychotherapy similarly influence emotion processing. Additionally, the present study investigated the mediating role of 5-HT_2A_ receptor stimulation on psychedelic-induced alterations of emotional empathy. Ketanserin has been previously shown to very effectively diminish all acute subjective responses to LSD (Preller et al., 2017; Holze et al., 2021), while not influencing plasma LSD concentrations (Holze et al., 2021). In the present study, ketanserin only weakly attenuated the LSD-induced increases in explicit and implicit empathy, and ratings remained elevated compared with placebo. This finding indicates that the empathogenic effects of LSD may not critically depend on 5-HT_2A_ receptor activation, unlike its subjective psychedelic effects, but could potentially be mediated by other receptors, including serotonin 5-HT_1_ receptors. A previous study that tested the 5-HT_1A_ agonist buspirone demonstrated the involvement of 5-HT_1A_ receptors in emotion processing (Bernasconi et al., 2015). LSD shows strong binding to 5-HT_1A_ receptors (Rickli et al., 2016), and this receptor is not blocked by ketanserin and could thus play a role in the LSD-induced enhancement of emotional empathy. Oxytocin has been shown to contribute to emotional processes, including empathy (Kosfeld et al., 2005; Di Simplicio et al., 2009; Ditzen et al., 2009; Hurlemann et al., 2010). Therefore, we also tested whether LSD increases circulating levels of oxytocin and whether this effect depends on 5-HT_2A_ receptor activation. LSD increased plasma oxytocin concentrations at the 200 μg dose (not measured for lower doses), consistent with a previous study (Schmid et al., 2015). In contrast, a lower dose of 100 μg LSD did not increase plasma oxytocin levels in another study (Holze et al., 2020). Furthermore, the LSD-induced increase in oxytocin in the present and previous studies by a high dose (Schmid et al., 2015) was only 1.25 to 3-fold higher compared with placebo, whereas MDMA increased plasma oxytocin levels 3 to 11-fold compared with placebo (Dolder et al., 2018; Holze et al., 2020). Thus, LSD may less effectively induce the release of oxytocin compared with MDMA. With regard to the mechanism of action, preclinical data indicate that MDMA induces its marked effects on oxytocin release via serotonin release and consecutive 5-HT_1_ receptor stimulation (Thompson et al., 2007; Hunt et al., 2011). In contrast, in the present study, ketanserin prevented the moderate LSD-induced increase in plasma oxytocin, indicating that the effects of LSD on the oxytocin system involve 5-HT_2A_ receptors, which is consistent with most other effects of LSD (Kraehenmann et al., 2017a; Kraehenmann et al., 2017b; Preller et al., 2017; Barrett et al., 2018; Preller et al., 2018; Holze et al., 2021). Thus, the mechanisms that result in oxytocin release may be distinct for MDMA and LSD and need further investigation. For example, the effects of LSD on oxytocin may be more indirect. LSD acutely induces marked alterations of perception and also moderately increases anxiety at 200 μg (Holze et al., 2021) while additionally elevating autonomic stimulation and plasma markers of serotonergic activity and stress, including circulating prolactin (Schmid et al., 2015) and glucocorticoids (Strajhar et al., 2016). Oxytocin may moderately increase in response to LSD-induced psychological stress. For example, oxytocin has been considered an anti-stress hormone (Rae et al., 2021). Thus, the oxytocin response might be triggered by subjective LSD-induced effects rather than by a direct pharmacological effect of LSD. Supporting this possibility, oxytocin levels did not increase when the subjective mind-altering effects of LSD were blocked with ketanserin. Instead, oxytocin levels only increased when subjective effects were also highly elevated. Taken together, the present findings indicate that LSD-induced empathogenic effects are neither mediated *via* oxytocin release nor via direct 5-HT_2A_ receptor activation and that the involvement of other factors and receptors in emotion processing is likely.

The present study has several strengths. The study used a pharmaceutically well-defined investigational drug product (Holze et al., 2019; Holze et al., 2021) and a highly valid double-blind, multi-dose, random-order design that included both inactive and “active” placebo in the form of low-dose LSD. Additionally, we assessed emotional empathy not only for a single dose but also for a range of doses and included an evaluation of the mediating role of a key target receptor of LSD within the same study and participants.

The present study also has limitations. We only included a small sample size of 16 participants. However, because we used a within-subjects design, we included data from 96 study sessions. Oxytocin was only measured for the highest 200 μg dose of LSD because the 100 μg dose showed no effect in previous studies. Because of the strong acute effects of LSD on cognitive performance and concentration (Schmid et al., 2015; Holze et al., 2020; Holze et al., 2021), the MET could not meaningfully be performed during the peak response to LSD. Instead, it was conducted 6 h after LSD administration when plasma LSD concentrations and subjective effects declined to approximately half-maximal levels. One possibility or likelihood was that the lower dose of LSD of 100 μg similarly enhanced empathy during the peak response at 3 h as the 200 μg at 6 h after LSD administration, but this was not tested. Thus, we cannot exclude the possibility that lower doses of LSD also significantly increase empathy. Furthermore, the present study included healthy subjects in a highly controlled laboratory setting, and the effects of LSD may be different in situations of uncontrolled recreational use or in therapeutic settings in patients (de Wit et al., 2021a; Schmid et al., 2021).

In conclusion, the present findings indicate that LSD enhances empathy, which is likely also beneficial for therapeutic application in patients. Psychedelics and MDMA have been shown to potentially trigger therapeutic processes and marked changes in personal attitudes and well-being after single-dose administration and with often lasting effects even in healthy subjects (Griffiths et al., 2008; Schmid and Liechti, 2018; de Wit et al., 2021b). These sometimes long-lasting effects are at least partially associated with alterations of mind and mystical-type experiences (Griffiths et al., 2008; Schmid and Liechti, 2018) and may also be linked to the acute experience of feeling strong emotional concern for others (i.e., emotional empathy) and a connection with other people (de Wit et al., 2021b). Remaining to be investigated is whether LSD also enhances empathy in patients with deficits in empathy and impairments in social functioning and whether such effects contribute to potential therapeutic effects of LSD.

## Data Availability

The raw data supporting the conclusion of this article will be made available by the authors, without undue reservation.
